# Supplementary material for: The Role of Oxygen in Avascular Tumor Growth
Source: PLoS One. 2016 Apr 18;11(4):e0153692. doi: 10.1371/journal.pone.0153692 (PMC4835055; doi:10.1371/journal.pone.0153692)
Supplement: S1 File — Fig A, Growth curve arising from oxygen model. The assumption of oxygen mediated growth gives rise to the classic sigmoidal growth curve. Fig B, Growth curves as a function of glucose availability. Lower glucose levels give rise to a modified growth curve as pm increases when glucose isn’t available. Despite pm changing by up to a factor of 10 under such circumstances, the growth curves under conditions of both ample glucose and glucose deficiency are quite similar. Fig C, Relative radii of spheroid sections with growth. For a spheroid of initial radii 20 μm and a = 7 × 10^−7^ m3 kg−1 s−1, the proliferating rim radius is the same as the spheroid radius. After r = rc, the proliferating radius falls relative to the anoxic radius rn which increases. At plateau, ro=rp3+rn33 and no further increase in volume is observed. Fig D, Varying removal rate of anoxic core. It is possible to also model situations where only a fraction of the anoxic core is lysed relative to cellular doubling time. This can be illustrated on the longest term projections for which we have experimentally determined OCR data—(a) HCT 116 and (b) SCC-25. This was fit to the data and a best-fit estimate of cellular doubling time td obtained, as illustrated. Fig E, Long term growth projections with different removal rates. Estimated projections for HCT 116 growth with time under different rate removal assumptions. Table A, Raw Seahorse oxygen consumption data for the HTC 116 cells used in this work. Table B, Raw Seahorse oxygen consumption data for the LS 147T cells used in this work. Table C, Raw Seahorse oxygen consumption data for the MDA-MB-468 cells used in this work. Table D, Raw Seahorse oxygen consumption data for the SCC-25 cells used in this work. Table E, Raw Seahorse oxygen consumption data for the MDA-MB-231 cells used in this work. Table F, Raw Seahorse oxygen consumption data for the U-87 cells used in this work. (PDF) [file pone.0153692.s001.pdf]

The role of oxygen in avascular tumor growth -Supplemental material

David Robert Grimes[1], Pavitra Kannan[1], Alan McIntyre[2], Anthony Kavanagh[3], Abul Siddiky[1], Simon Wigfield[2], Adrian Harris[2], Mike Partridge[1]

1 Cancer Research UK/MRC Oxford Institute for Radiation Oncology, Gray Laboratories, University of Oxford, Old Road Campus, Oxford, OX3 7DQ
2 The Weatherall Institute for Molecular Medicine, University of Oxford, John Radcliffe Hospital/Headley Way, Oxford, OX3 9DS
3 Advanced Technology Development Group, Department of Oncology, University of Oxford, Old Road Campus Research Building, Oxford, OX3 7DQ
*** davidrobert.grimes@oncology.ox.ac.uk**

Supporting Information

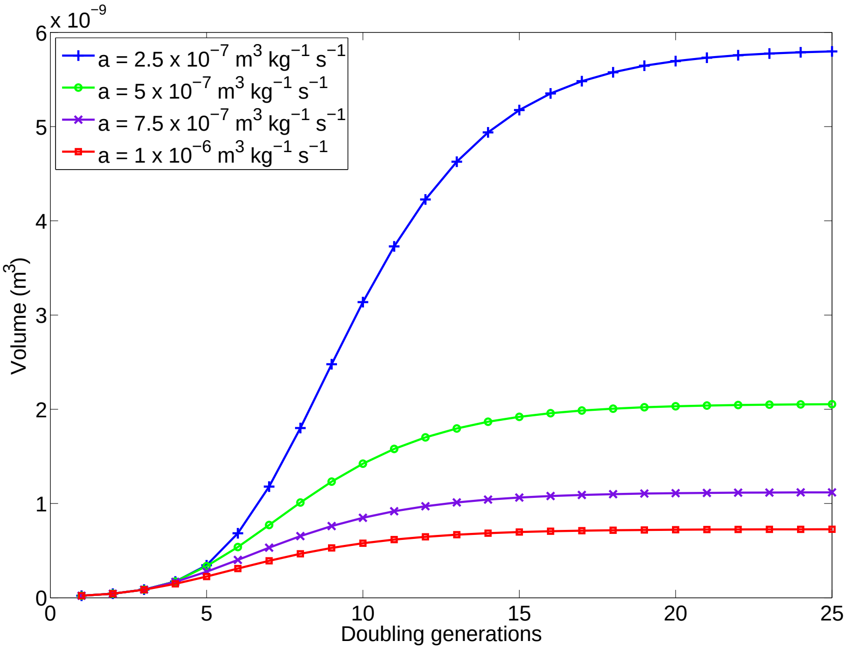

S1 Fig A: Sigmoidal curves from this model. Projected sigmoidal curves resulting from oxygen model for a spheroid of initial radius 50 μm after 25 doubling generations. Plateau volume and grow characteristics are heavily influence by oxygen consumption rate.

S1 Fig A

Growth curve arising from oxygen model The assumption of oxygen mediated growth gives rise to the classic sigmoidal growth curve.

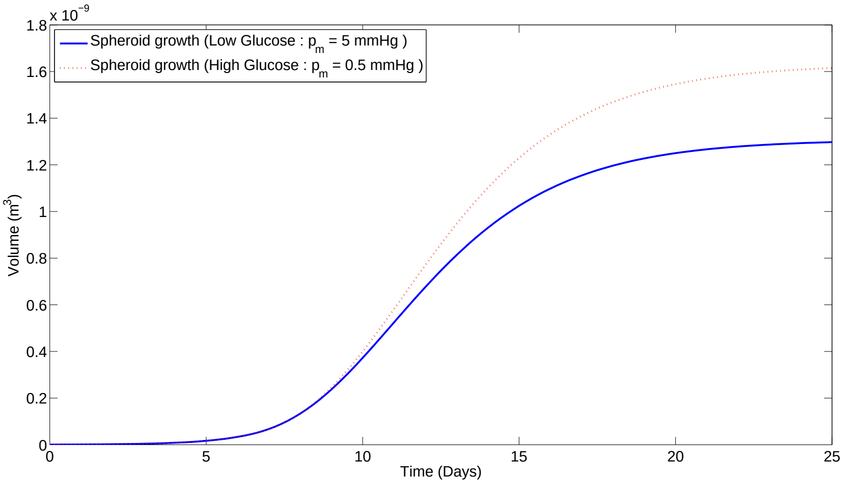

S1 Fig B: Growth curves as a function of glucose availability. Two hypothetical spheroids with doubling time of 1 day and diffusion limit of 250 μm (19.2 mmHg /s) with an initial radius of 50 μm. The spheroid with an ample supply of glucose can reach a greater theoretical plateau volume than the low glucose spheroid.

S1 Fig B

Growth curves as a function of glucose availability Lower glucose levels give rise to a modified growth curve as p_m increases when glucose isn't available. Despite p_m changing by up to a factor of 10 under such circumstances, the growth curves under conditions of both ample glucose and glucose deficiency are quite similar.

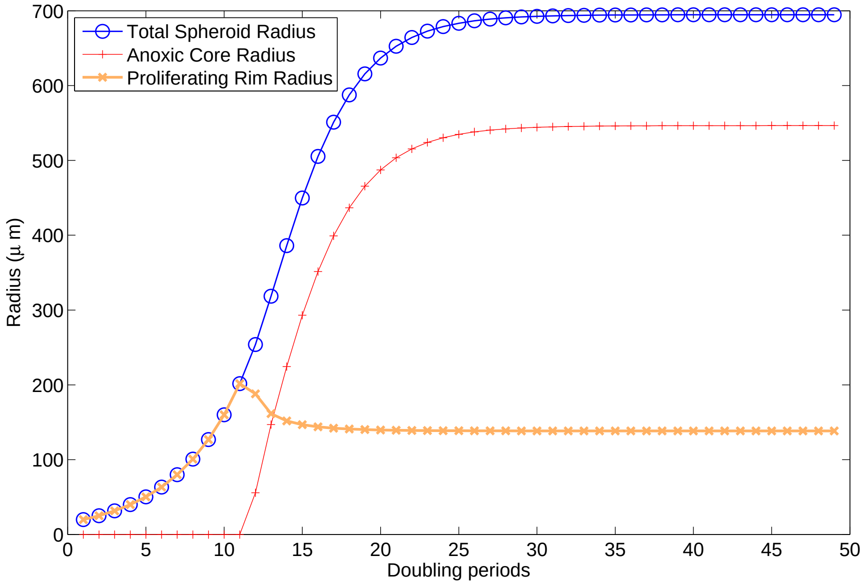

S1 Fig C: Relative radii of spheroid sections with growth. For a spheroid of initial radii 20 μm and $a = 7 \times 10^{-7}$ m^3 kg^{-1} s^{-1}, the proliferating rim radius is the same as the spheroid radius. After $r = r_c$, the proliferating radius falls relative to the anoxic radius r_n which increases. At plateau, $r_o = \sqrt[3]{r_p^3 + r_n^3}$ and no further increase in volume is observed.

S1 Fig C

Relative radii of spheroid sections with growth.

S1 Fig D

Incomplete removal of anoxic core cell debris. Using experimentally derived OCR estimates for given cell lines, we can also project growth curves if smaller fractions of the core are reduced, as discussed in the main text. This has been fit to the data with the longest range available (HCT 116 and SCC-25). However, these fits are not as good as fits under the assumption of total core removal. In both cases, a simple linear fit gives an $R^2 = 0.97$. This good linear fit is expected, as from the OCR calculations outlined in the paper spheroids in the data set have $r > r_s$ and are below plateau volume, and so are expected to display quasi-linear growth phase between r_s and plateau phase. Projected plateau radius / volume for HCT 116 is 606 μm / 9.31 $\times 10^{-10}$ m^3 and 804μm / 2.18 $\times 10^{-9}$ m^3 for SCC-25.

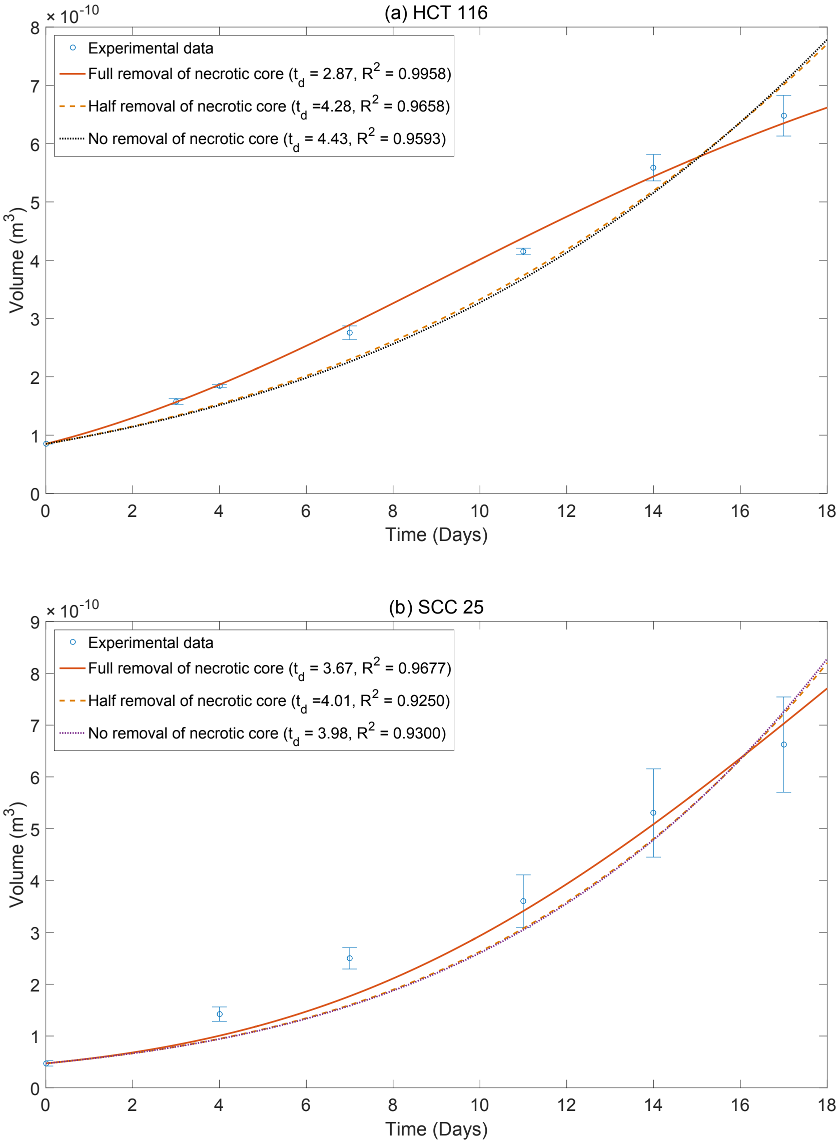

S1 Fig D: Varying removal rate of anoxic core It is possible to also model situations where only a fraction of the anoxic core is lysed relative to cellular doubling time. This can be illustrated on the longest term projections for which we have experimentally determined OCR data - (a) HCT 116 and (b) SCC-25. This was fit to the data and a best-fit estimate of cellular doubling time t_d obtained, as illustrated.

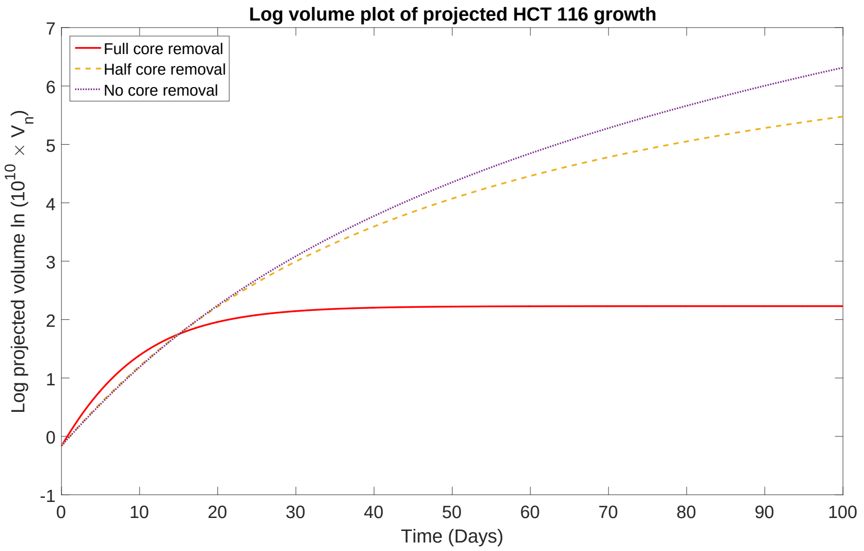

S1 Fig E: Long term growth projections with different removal rates Estimated projections for HCT 116 growth with time under different rate removal assumptions.

S1 Fig E

Under the assumption of full core removal with experimentally derived OCR, HCT 116 spheroids are expected to plateau when a radius of 606 μm has been obtained. By contrast, with half-core removal per time step spheroids would obtain a huge plateau radius of 3mm after 150 doubling generations, or 642 days in this example. For zero core removal, no plateau is ever reached and volume increases rapidly. Long term projected growth without complete core removal deviates significantly from experimental observation, indicating full core removal best describes observed data.

Oxygen consumption rate Tables

Seahorse oxygen consumption rate (OCR) data for all cell lines used in this study. (See details in main text).

Table A: HCT 116 OCR Data

Number of Cells	S_H (pM / min)	Number of repeats
5000	14.60358 ± 5.31686	4
10000	32.72503 ± 8.73702	4
15000	55.43225 ± 7.05496	4
20000	88.9986 ± 8.90586	4
30000	143.0673 ± 6.58889	4
40000	198.3031 ± 21.26691	4
50000	242.994 ± 25.11013	4
60000	322.389 ± 7.91631	4
70000	396.6938 ± 14.0226	4
80000	458.3384 ± 34.44188	4
100000	546.1055 ± 9.46332	4

Table B: LS 147T OCR Data

Number of Cells	S_H (pM / min)	Number of repeats
5000	7.09419 ± 3.45938	4
10000	15.88206 ± 9.0363	4
15000	24.99787 ± 3.55027	4
20000	46.92665 ± 9.56276	4
30000	79.78763 ± 15.55025	4
40000	116.5803± 19.97117	4
50000	179.5878± 5.05769	4
60000	230.968 ±8.47642	4
70000	258.4062 ± 17.36056	4
80000	342.1105± 19.86468	4
100000	397.8702± 35.65412	4

Table C: MDA-MB-468 OCR Data

Number of Cells	S_H (pM / min)	Number of repeats
5000	17.23148 ± 3.84692	4
10000	41.59406 ± 4.9002	4
15000	72.49963 ± 8.32524	4
20000	93.76402 ± 20.3249	4
30000	143.9684 ± 8.9862	4
40000	211.0183 ± 10.3962	4
50000	260.7484 ± 10.61333	4
60000	317.5164 ± 23.61896	4
70000	387.3681 ± 21.63372	4
80000	461.7557 ± 35.65298	4
100000	537.4339 ± 1.02598	4

Table D: SCC-25 OCR Data

Number of Cells	S_H (pM / min)	Number of repeats
5000	13.3393 ± 1.76108	4
10000	31.45891 ± 7.28148	4
15000	39.33114 ± 10.90834	4
20000	59.46781 ± 10.00824	4
30000	93.00727 ± 20.16541	4
40000	130.6053 ± 14.78212	4
50000	148.0849 ± 20.40247	4
60000	167.1917 ± 10.63227	4
70000	188.6037 ± 12.67655	4
80000	180.3511 ± 45.50524	4
100000	187.6634 ± 19.00578	4

Table E: MDA-MB-231 OCR Data

Number of Cells	S_H (pM / min)	Number of repeats
1000	1.70463 ± 3.65947	6
2500	6.35964 ±2.60227	8
5000	16.81834 ± 5.04472	8
10000	34.74601 ± 6.30807	8
12500	46.8847 ± 3.39043	8
15000	56.5616 ± 6.87928	8
17500	74.76814 ± 5.37414	8
20000	77.34116 ± 6.36223	8
25000	94.04961 ± 8.57692	8
30000	122.3539 ± 12.20188	8
40000	132.1118 ± 12.24266	8
50000	160.2979 ± 6.67291	6

Table F: U-87 OCR Data

Number of Cells	S_H (pM / min)	Number of repeats
2500	2.23223 ± 2.14217	4
5000	15.9857 ± 9.99255	4
10000	20.29126 ± 6.26257	4
15000	22.45436 ± 4.44754	4
20000	24.52205 ± 5.2108	4
25000	23.42007 ± 6.31214	4
30000	23.25213 ± 4.89798	4
40000	19.59612 ± 8.74149	4
50000	19.38005 ± 3.62698	4
60000	25.07353 ± 4.82978	4
80000	20.96915 ± 3.18112	4
100000	38.79394 ± 5.85128	4
